# Analytical and Physical Investigation on Source Resistance in In*_x_*Ga*_1−x_*As Quantum-Well High-Electron-Mobility Transistors

**DOI:** 10.3390/mi14020439

**Published:** 2023-02-12

**Authors:** Ji-Hoon Yoo, In-Geun Lee, Takuya Tsutsumi, Hiroki Sugiyama, Hideaki Matsuzaki, Jae-Hak Lee, Dae-Hyun Kim

**Affiliations:** 1 School of Electronic and Electrical Engineering, Kyungpook National University (KNU), Daegu 41566, Republic of Korea; 2 NTT Device Technology Laboratories, Atsugi-shi 243-0198, Japan

**Keywords:** source resistance, TLM, In*_x_*Ga*_1−x_*As, HEMT

## Abstract

We present a fully analytical model and physical investigation on the source resistance (*R_S_*) in In*_x_*Ga*_1−x_*As quantum-well high-electron mobility transistors based on a three-layer TLM system. The *R_S_* model in this work was derived by solving the coupled quadratic differential equations for each current component with appropriate boundary conditions, requiring only six physical and geometrical parameters, including ohmic contact resistivity (*ρ_c_*), barrier tunneling resistivity (*ρ_barrier_*), sheet resistances of the cap and channel regions (*R_sh_cap_* and *R_sh_ch_*), side-recessed length (*L_side_*) and gate-to-source length (*L_gs_*). To extract each model parameter, we fabricated two different TLM structures, such as *cap-TLM* and *recessed-TLM*. The developed *R_S_* model in this work was in excellent agreement with the *R_S_* values measured from the two TLM devices and previously reported short-*L_g_* HEMT devices. The findings in this work revealed that barrier tunneling resistivity already played a critical role in reducing the value of *R_S_* in state-of-the-art HEMTs. Unless the barrier tunneling resistivity is reduced considerably, innovative engineering on the ohmic contact characteristics and gate-to-source spacing would only marginally improve the device performance.

## 1. Introduction

The evolving sixth-generation (6G) wireless communication technologies demand higher operating frequencies of approximately 300 GHz with data rates approaching 0.1 Tbps [1,2]. To meet this urgent requirement, transistor technologies must be engineered to sustain the evolution of digital communication systems, guided by Edholm’s law [3]. Among various transistor technologies, indium-rich In*_x_*Ga*_1−x_*As quantum-well (QW) high-electron-mobility transistors (HEMTs) on InP substrates have offered the best balance of current-gain cutoff frequency (*f_T_*) and maximum oscillation frequency (*f_max_*), and the lowest noise figure characteristics in the sub-millimeter-wave region [4,5,6,7,8]. These transistors adopt a combination of *L_g_* scaling down to sub-30 nm, enhancement of the channel carrier transport by incorporating the indium-rich channel design, and reduction of all parasitic components.

Among various parasitic components, it is imperative to minimize the source resistance (*R_S_*) itself to fully benefit from the superior intrinsic performance of the In*_x_*Ga*_1−x_*As QW channel [9,10], demanding an analytical and physical model for the source resistance. Considering state-of-the-art In*_x_*Ga*_1−x_*As HEMT technologies [11,12,13,14], source and drain contacts have been created with a non-alloyed metal stack of Ti/Pt/Au with a source-to-drain spacing (*L_ds_*) between 1 μm and 0.5 μm. Historically, *R_S_* is minimized by reducing the ohmic contact resistivity (*ρ_c_*) [15] and shrinking the gate-to-source spacing (*L_gs_*) using da self-aligned gate architecture [16,17]. However, it is very challenging to reduce *R_S_* to below 100 Ω·μm, because of the tunneling resistance component between the heavily doped In*_0.53_*Ga*_0.47_*As capping layer and the In*_x_*Ga*_1−x_*As QW channel layer. To understand the limit of *R_S_* in HEMTs in an effort to further reduce *R_S_*, a sophisticated and comprehensive model must be developed for *R_S_* in state-of-the art HEMTs, rather than the simple lumped-elements-based one-layer model [18,19].

Previously, two-layer system-based *R_S_* model was developed by Feuer [20], which was applicable to alloyed ohmic contact structures with two different contact resistances: one was associated with a heavily doped GaAs capping layer and the other with a undoped GaAs QW channel layer. In this letter, we present a fully analytical and physical model for *R_S_* in advanced HEMTs, requiring only six physical and geometrical parameters. The model considers three different regions: (i) a one-layer transmission-line model (TLM) for the side-recess region, (ii) an analytical TLM for the access region and (iii) an analytical three-layer TLM for the source electrode region, to accurately predict a value of *R_S_* in a given HEMT structure and identify dominant components to further minimize *R_S_*. To do so, we proposed and fabricated two different types of TLM structures to experimentally extract each component of *R_S_*. The analytical model proposed in this work is in excellent agreement with the measured values of *R_S_* from the fabricated *r-TLMs*, as well as recently reported advanced HEMTs. Most importantly, findings in this work reveal that the *ρ_barrier_* is a bottleneck for further reductions of *R_S_* in advanced HEMTs.

## 2. Analytical Model for *R_S_*

**Figure 1:**
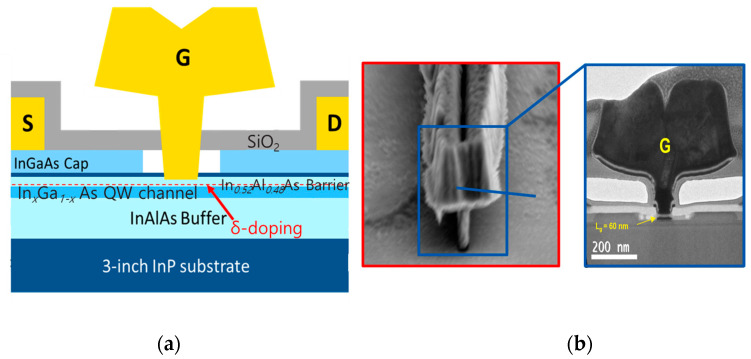
(**a**) Cross-sectional schematic, and (**b**) TEM images of advanced In*_x_*Ga*_1−x_*As QW HEMTs [4].

Figure 1a–c show the cross-sectional schematic and TEM images of advanced In*_x_*Ga*_1−x_*As QW HEMTs on an InP substrate [4]. They adopt non-alloyed S/D ohmic contacts such as a metal stack of Ti/Pt/Au with contact resistance (*R_C_*) values between 10 Ω·μm and 20 Ω·μm. Carrier transfer from the cap to channel replies on a tunneling mechanism via an In*_0.52_*Al*_0.48_*As barrier layer. To model *R_S_*, a comprehensive transport mechanism from the source ohmic electrode to the In*_x_*Ga*_1−x_*As QW channel via the In*_0.53_*Ga*_0.47_*As cap and In*_0.52_*Al*_0.48_*As barrier layers must be considered in a distributed manner.

**Figure 2:**
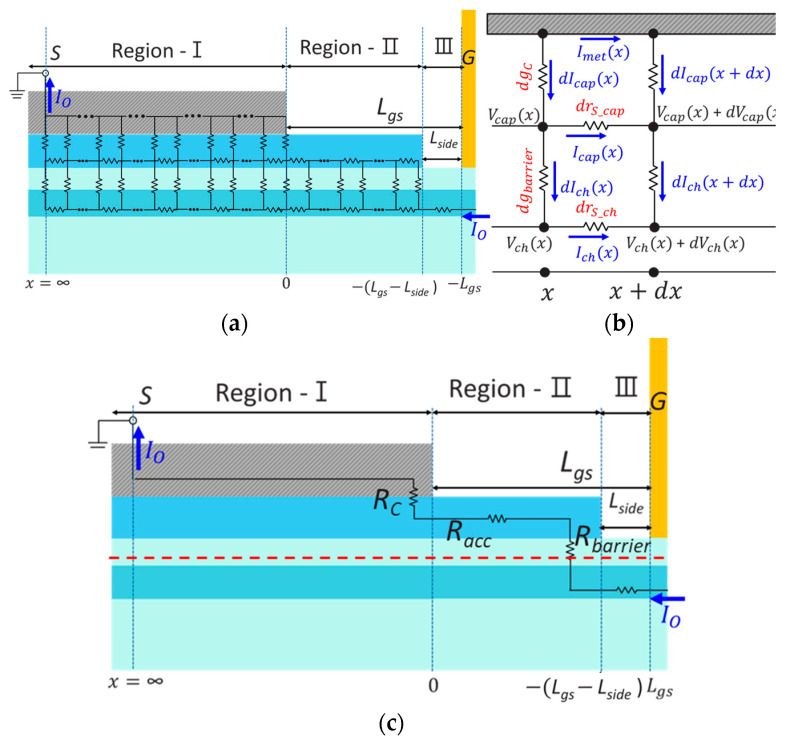
(**a**) Equivalent circuit model of the source structure in the advanced HEMTs, (**b**) differential segment from *x* to *x* + *dx*, and (**c**) lumped-elements based one-layer model.

Figure 2a illustrates a complete distributed equivalent circuit model for *R_S_*, comprising three regions. One is the source ohmic electrode region (Region-I), where the electrons are injected from the ohmic metal to the In*_0.53_*Ga*_0.47_*As cap and then to the In*_x_*Ga*_1−x_*As QW channel through the In*_0.52_*Al*_0.48_*As barrier, which is governed by a three-layer TLM system. Another is the source access region (Region-II), where the electron transfer mechanism is governed by a cap-to-channel two-layer TLM system with transfer length (*L_T_barrier_*) given by $\sqrt{\rho_{barrier}/\left(R_{sh\_ch}+R_{sh\_cap}\right)}$. The other is the side-recessed region (Region-III), where a simple one-layer model works. In comparison, lumped-elements based one-layer model is shown in Figure 2c [18,19].

**Figure 3:**
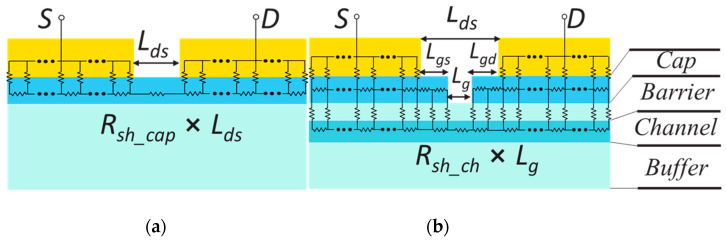
Cross-sectional schematic of *cap-TLM* (**a**) and recessed-TLM (*r-TLM*) (**b**).

Next, let us derive a fully analytical and physical expression for *R_S_*. Given the coordinate system in Figure 3a, *R_S_* can be determined by *V_ch_*(*x* = −*L_gs_*)/*I_O_* from Ohm’s law, and then the problem is how to express each current component as a function of *x* such as *I_ch_*(*x*), *I_cap_*(*x*), and *I_met_*(*x*). In a given segment as highlighted in Figure 3b, we can define a differential contact conductance as *dg_c_* = (*W_g_*/*ρ_c_*) *dx*, a differential barrier conductance as *dg_barrier_* = (*W_g_*/*ρ_barrier_*) *dx*, a differential lateral cap resistance as *dr_s_cap_* = (*R_sh_cap_*/*W_g_*) *dx* and a differential lateral channel resistance as *dr_s_ch_* = (*R_sh_ch_*/*W_g_*) *dx*. At location *x*, Kirchhoff’s current and voltage laws yield, respectively

(1) $$\frac{d^{2}I_{met}\left(x\right)}{dx^{2}}=\left[R_{met}\cdot I_{met}\left(x\right)-R_{sh\_ch}\cdot I_{cap}\left(x\right)\right]\rho_{c}^{-1}$$

(2) $$\frac{d^{2}I_{ch}\left(x\right)}{dx^{2}}=\left[R_{sh\_ch}\cdot I_{ch}\left(x\right)-R_{sh\_cap}\cdot I_{cap}\left(x\right)\right]\rho_{barrier}^{-1}$$

(3) $$I_{cap}=I_{O}-I_{met}-I_{ch}$$

These are coupled quadratic differential equations for three current components (*I_ch_*(*x*), *I_cap_*(*x*) and *I_met_*(*x*)). From the general solution for these differential equations with existing six boundary conditions (listed in Table 1), we obtain an analytical expression for *I_ch_*(*x*), *I_cap_*(*x*), and *I_met_*(*x*) for both regions, as written in Table 1. The expression for *V_ch_*(*x* = −*L_gs_*) can then be derived. Although there are several ways to express *V_ch_*(*x* = −*L_gs_*), it is useful to focus on the total voltage drop across the In*_x_*Ga*_1−x_*As QW channel from *x* = −*L_gs_* to *x* = ∞ in this work. From this,

(4) $$V_{ch}(x=-L_{gs})=\int I_{ch}\left(x\right)\cdot dr_{S\_ch}\;dx$$

The source resistance, defined as *V_ch_*(*x* = 0)/*I_O_*, is

(5) $$R_{S}=\frac{V_{ch}\left(x=-L_{gs}\right)}{I_{O}}=\frac{R_{sh\_ch}}{W_{g}I_{O}}\int_{-L_{gs}}^{\infty }I_{ch}\left(x\right)dx$$

(6) $$R_{S}\cdot W_{g}=[\frac{\sqrt{2}\cdot C_{1}\cdot \sqrt{\rho_{C}\cdot \rho_{barrier}}}{\rho_{C}\cdot \left(R_{sh\_ch}+R_{sh\_cap}\right)+\rho_{barrier}\cdot R_{sh\_cap}-\eta }+\frac{\sqrt{2}\cdot C_{1}\cdot \sqrt{\rho_{C}\cdot \rho_{barrier}}}{\rho_{C}\cdot \left(R_{sh\_ch}+R_{sh\_cap}\right)+\rho_{barrier}\cdot R_{sh\_cap}+\eta }\;+\frac{\left(C_{3}-C_{4}\right)\cdot \left(1-e^{-L_{gs}/L_{T\_barrier}}\right)}{L_{T\_barrier}}+\frac{R_{sh\_cap}\cdot L_{gs}}{\rho_{barrier}\cdot L_{T\_barrier}{}^{2}}]+R_{sh\_ch}\cdot L_{side}$$

(7) $$\eta =\sqrt{\rho_{c}{}^{2}(R_{sh\_ch}{}^{2}+R_{sh\_cap}{}^{2})+2R_{sh\_ch}\;R_{sh\_cap}\rho_{c}\left(\rho_{c}-\rho_{barrier}\right)+R_{sh\_cap}{}^{2}\rho_{barrier}\left(2\rho_{c}+\rho_{barrier}\right)}$$

(8) $$L_{T\_barrier}=\sqrt{\frac{\rho_{barrier}}{R_{sh\_ch}+R_{sh\_cap}}}$$

Overall, *R_S_* depends on the ohmic contact resistivity, the sheet resistances of the cap and QW channel layers, the barrier tunneling resistivity, and the length of the gate-to-source region and side-recessed regions.

## 3. Experimental Results and Discussion 

Two types of TLM structures were fabricated, as shown in Figure 3: the cap-only TLM structure (*cap-TLM*, (a)) to evaluate the contact characteristics of the non-alloyed ohmic metal stack, and the recessed TLM structure (*r-TLM*, (b)) which is identical to the real device without a Schottky gate electrode. Details on the epitaxial layer design and device processing were reported in our previous paper [4]. All device processing was conducted on a full 3-inch wafer with an i-line stepper to ensure fine alignment accuracy within 0.05 μm. In the *r-TLM*, we varied *L_g_* from 40 μm to 0.5 μm and *L_gs_* from 10 μm to 0.2 μm. In this way, the split of *L_g_* yielded the sheet resistance of the QW channel (*R_sh_ch_*) from the linear dependence, and the source resistance (*R_S_*) from the y-intercept at a given *L_gs_*. Lastly, we investigated the dependence of *R_S_* on *L_gs_* in detail.

**Figure 4:**
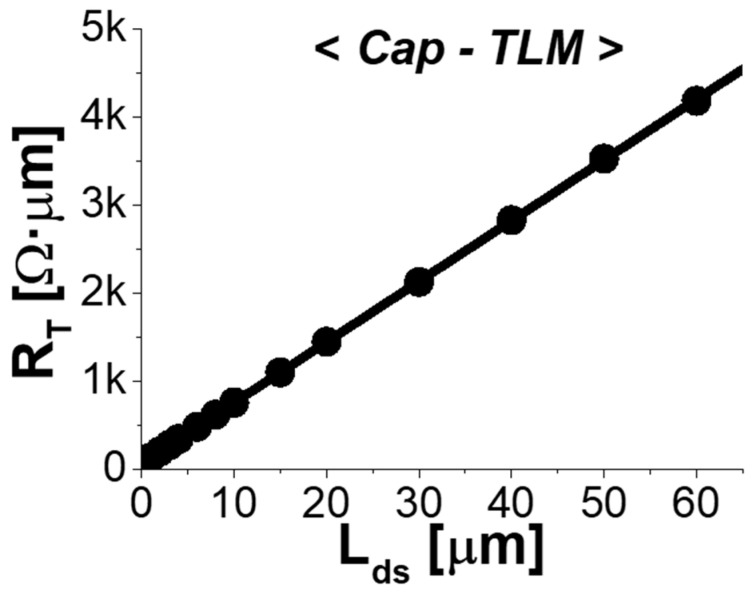
Measured total resistance (*R_T_*) as a function of *L_ds_* for *cap-TLM*.

**Figure 5:**
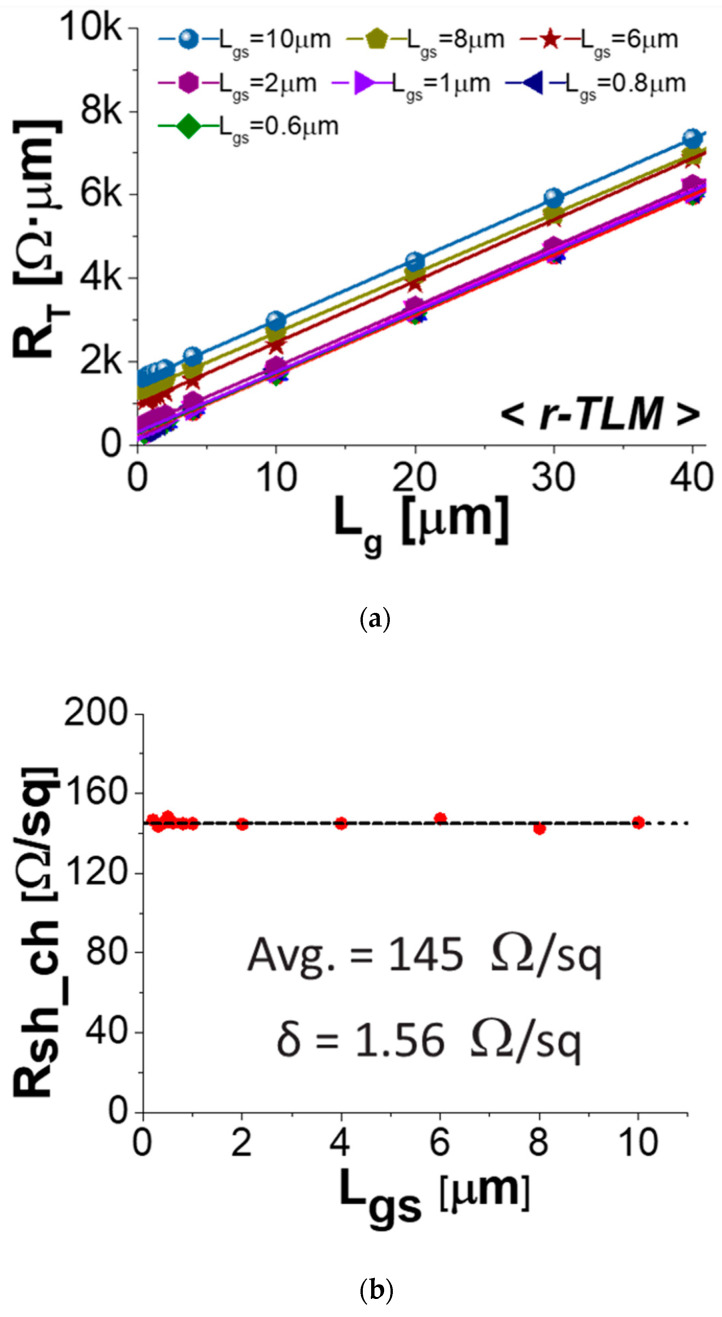
(**a**) Measured total resistance (*R_T_*) as a function of *L_g_* for *r-TLM* with various dimensions of L*_gs_* from 10 μm to 0.2 μm, and (**b**) the extracted sheet resistance of the In*_x_*Ga*_1−x_*As QW channel (*R_sh_ch_*) as a function of *L_gs_*.

**Figure 6:**
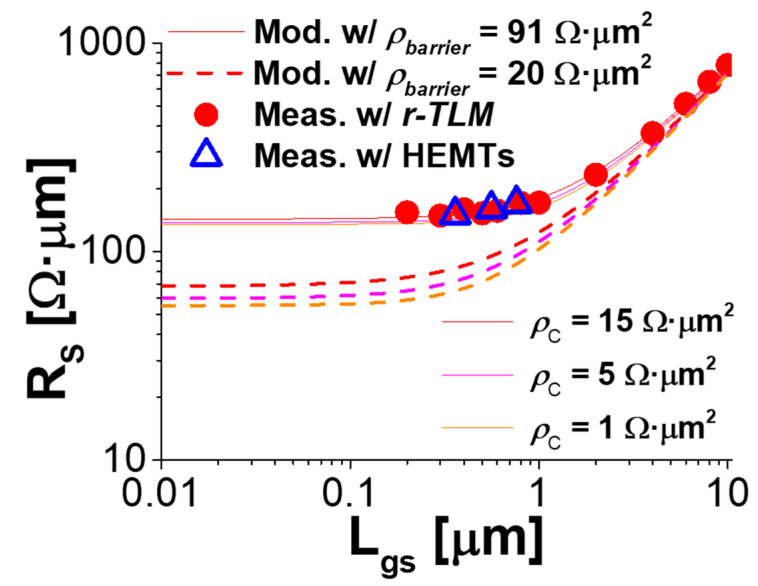
Comparison of the modeled and measured *R_S_* against *L_gs_* in the *log-log* scale.

Figure 4 plots the measured total resistance (*R_T_*) against *L_ds_,* which corresponds to the length between the edge of source and the edge of drain. for the fabricated *cap-TLM* structures. This yielded values of *R_sh_cap_* = 131 Ω/sq, *R_C_* = 32 Ω·μm, *L_T_cap_* = 0.34 μm and *ρ_c_* = 15 Ω·μm^2^, with an excellent correlation coefficient of 0.99999. Figure 5a plots the measured *R_T_* against *L_g_* for the *r-TLM* structures with various dimensions of *L_gs_* from 10 μm to 0.2 μm. When *L_g_* was long enough, each *r-TLM* device yielded approximately the same slope for all *L_gs_* with excellent correlation coefficient. This is plotted in Figure 5b with averaged *R_sh_ch_* = 145 Ω/sq and excellent ∆(*R_sh_ch_*) = 1.56 Ω/sq, confirming that the In*_0.8_*Ga*_0.2_*As QW channel sheet resistance was independent of *L_gs_*. Because we designed the symmetrical *L_gs_* and *L_gd_*, half of the *y*-intercept from Figure 6a corresponded exactly to *R_S_*. In analyzing *r-TLM* structures with various *L_gs_*, values of the correlation coefficient were also greater than 0.999, increasing the credibility of the overall TLM analysis.

Figure 6 plots the measured *R_S_* (filled symbols) from the *r-TLM* analysis against *L_gs_*, as well as the projected *R_S_* (line) from Equation (5) with the model parameters of *ρ_barrier_* = 91 Ω·μm^2^ and others directly from the *cap-TLMs* and *r-TLMs*. Additionally, the open symbols in Figure 6 came from the *R_S_* extracted directly from the reported HEMTs [4] using the gate-current injection technique [21]. There are two points to identify in Figure 6. First, all of the measured *R_S_* characteristics were explained by the modeled *R_S_*. Second, *R_S_* was linearly proportional to *L_gs_* for *L_gs_* > 1 μm, where its slope was 69 Ω/sq. Interestingly, this was similar to the parallel connection of *R_sh_cap_* and *R_sh_ch_*. However, this linear dependence of *R_S_* on *L_gs_* was no longer valid for *L_gs_* < 1 μm and, most importantly, the measured *R_S_* eventually saturated to approximately 123 Ω·μm even with *L_gs_* approaching 0. Our model clearly indicated that this was because of the barrier tunneling resistivity. The saturation of *R_S_* in *L_gs_* = 0 was because the necessary lateral length for the cap-to-channel tunneling was supplied by its equivalent transfer length from the leading edge of the source metal contact (−*L_T_barrier_* < *x* < 0) in Region-I. 

Finally, let us discuss how to further reduce *R_S_* with the *R_S_* model proposed in this work. The three solid lines in Figure 6 are the model projections of *R_S_* with the ohmic contact resistivity improve from 15 Ω·μm^2^ (present) to 1 Ω·μm^2^. Surprisingly, *R_S_* would not be minimized even with a significant reduction in *ρ_c_* and *L_gs_* because of the *ρ_barrier_*. Alternatively, the three dashed lines in Figure 6 are from the same model projection, but with *ρ_barrier_* = 20 Ω·μm^2^. Note that a reduction in the *ρ_barrier_* is important; in consequence, the projected *R_S_* would be significantly scaled down to 70 Ω·μm and below. Under this circumstance, *R_S_* could then be further reduced by the improved ohmic contact characteristics and the reduction of *L_gs_*.

## 4. Conclusions

A fully analytical and physical investigation on *R_S_* in advanced In*_x_*Ga*_1−x_*As QW HEMTs was carried out with a three-layer TLM system. Analytical solutions to the three current components (source metal, cap, and channel) along the selected coordinate system with appropriate boundary conditions were produced. The proposed *R_S_* model in this work required only six physical and geometrical parameters (*ρ_c_*, *ρ_barrier_*, *R_sh_cap_*, *R_sh_ch_*, *L_side_* and *L_gs_*), yielding excellent agreement with the *R_S_* values measured from the two TLM devices and previously reported In*_x_*Ga*_1−x_*As QW HEMTs. The developed model in this work was capable of explaining the saturation behavior of *R_S_* for *L_gs_* < 1 μm, which was due to the *ρ_barrier_*. Therefore, one must pay a more careful attention to cut down the *ρ_barrier_* to further minimize *R_S_* in future HEMTs.

## Figures and Tables

**Table 1:** Six boundary conditions, the general solution for three current components, and their corresponding eigenvalues and eigenvectors.

BCs	$I_{ch}$$=I_{O}$ for *x =* −*L_gs_*	$I_{met}\;$= 0 for −*L_gs_* < *x* < 0	$I_{met}$ = $I_{O}$ for *x* = ∞
	$I_{cap}$(x=*0*^−^$)=I_{cap}$$(x=0^{+}$)	$I_{ch}$(x=*0*^−^$)=I_{ch}$$(0^{+}$)	$\frac{dI_{ch}(x=0^{-})\;}{dx}=-\frac{dI_{ch}\left(x=0^{+}\right)}{dx}$
Region-I	$\left[\begin{array}{c} I_{met}\\ I_{ch} \end{array}\right]=\left[\begin{array}{c} v_{11}\\ v_{12} \end{array}\right]C_{1}exp\left(-\sqrt{\lambda_{1}}x\right)+\left[\begin{array}{c} v_{21}\\ v_{22} \end{array}\right]C_{2}exp\left(-\sqrt{\lambda_{2}}x\right)+\left[\begin{array}{c} I_{O}\\ 0 \end{array}\right]$ $I_{cap}=I_{O}-I_{met}-I_{ch}$	Region-II	$I_{cap}=C_{3}exp\left(\frac{-x}{L_{T\_barrier}}\right)+C_{4}exp\left(\frac{x}{L_{T\_barrier}}\right)+\frac{R_{sh\_ch}}{R_{sh\_cap}+R_{sh\_ch}}I_{O}$ $I_{ch}=I_{O}-I_{cap}$
−$\;\lambda_{1}$ & $\lambda_{2}$ are eigenvalues of $A=\left[\begin{array}{cc} \left(\frac{R_{sh\_cap}}{\rho_{c}}\right) & \left(\frac{R_{sh\_cap}}{\rho_{c}}\right)\\ \left(\frac{R_{sh\_cap}}{\rho_{barrier}}\right) & \left(\frac{R_{sh\_cap}+R_{sh\_cap}}{\rho_{barrier}}\right) \end{array}\right]$ −$\left[\begin{array}{c} v_{11}\\ v_{12} \end{array}\right]\;\&$ $\left[\begin{array}{c} v_{21}\\ v_{22} \end{array}\right]$ are their corresponding eigenvectors		$\left[\begin{array}{c} C_{1}\\ C_{2}\\ \begin{array}{c} C_{3}\\ C_{4} \end{array} \end{array}\right]={\left[\begin{array}{cccc} 0 & 0 & exp\left(\frac{L_{gs}}{L_{T\_barrier}}\right) & exp\left(\frac{-L_{gs}}{L_{T\_barrier}}\right)\\ v_{12} & v_{22} & 1 & 1\\ v_{11} & v_{21} & 0 & 0\\ -\sqrt{\lambda_{1}}v_{12} & -\sqrt{\lambda_{2}}v_{22} & -\frac{1}{L_{T\_barrier}} & \frac{1}{L_{T\_barrier}} \end{array}\right]}^{-I}\left[\begin{array}{c} -\frac{R_{sh\_ch}\times I_{O}}{R_{sh\_cap}+R_{sh\_ch}}\\ I_{O}\left(1-\frac{R_{sh\_ch}}{R_{sh\_cap}+R_{sh\_ch}}\right)\;\\ \begin{array}{c} -I_{O}\\ 0 \end{array} \end{array}\right]$	

## Data Availability

The data presented in this study are available on request from the corresponding author.
